# Chuangling Ye mitigates diabetic foot ulcer through suppressing keratinocyte ferroptosis via inhibiting ACSS2/ACSL4 axis

**DOI:** 10.1186/s13020-026-01435-8

**Published:** 2026-06-08

**Authors:** Xiao Feng, Nan Yi, Cunyu Zhang, Liang Tang, Xindong Yin, Ya Zhao, Taiyang Zhu, Jianhua Zhu, Qian Feng, Weiwei Chen, Yawen Xia, Chaoqun Ma

**Affiliations:** 1 Department of General Surgery, Affiliated Hospital of Nanjing University of Chinese Medicine, Nanjing, 210029 China; 2 The First Clinical Medical College, Nanjing University of Chinese Medicine, Nanjing, 210023 China; 3 Department of Vascular Surgery, Affiliated Hospital of Nanjing University of Chinese Medicine, Nanjing, 210029 China; 4 Key Laboratory of Acupuncture and Medicine Research of Ministry of Education, Nanjing University of Chinese Medicine, Nanjing, 210023 China; 5 College of Acupuncture-Moxibustion and Tuina, Nanjing University of Chinese Medicine, Nanjing, 210023 China; 6 Nanjing Jiangning Hospital of Chinese Medicine, Nanjing, 211100 China

**Keywords:** Chuangling Ye, Diabetic foot ulcer, Ferroptosis, Acyl-CoA synthetase short-chain family member 2, Acyl-CoA synthetase long chain family member 4

## Abstract

**Background:**

Diabetic foot ulcers (DFU) is a serious complication of diabetes, and keratinocyte ferroptosis has been shown to accelerate its pathological progression. Chuanglin Ye (CLY), widely used in clinical practice for the treatment of DFU, has been reported to exert anti-inflammation effects and promote skin regeneration. However, its pharmacological mechanisms on DFU remain unclear.

**Methods:**

The DFU rats were treated with CLY (0.225 and 0.45 g/mL) for 21 days to evaluate its therapeutic efficacy. The chemical composition of CLY was analyzed using UPLC-MS/MS. Network pharmacology, western blot, and immunohistochemical staining were performed to assess the anti-ferroptosis effects of CLY. To validate the protective role of CLY against ferroptosis, RSL3, a ferroptosis inducer, was applied in this study. Subsequently, HaCaT cells were exposed to advanced glycation end products (AGEs) to establish an in vitro model of keratinocyte ferroptosis, further confirming the inhibitory effects of CLY on ferroptosis. RNA sequencing was conducted to elucidate the molecular mechanisms underlying CLY-mediated protection against keratinocyte ferroptosis. The functional involvement of the ACSS2/ACSL4 axis was verified through plasmid-mediated gene overexpression in vitro. Molecular docking analysis was employed to identify potential bioactive compounds in CLY that target ACSS2.

**Results:**

CLY administration significantly accelerated wound healing in DFU rats. Network pharmacology analysis indicated the regulation of ferroptosis underlies the therapeutic effect of CLY against DFU, which was further supported by its ability to ameliorate lipid peroxidation and iron overload. In contrast, the ferroptosis inducer RSL3 attenuated the protective effects of CLY in DFU rats. In vitro, CLY markedly suppressed AGEs-induced lipid peroxidation and excessive ROS level. Mechanistically, RNA-seq analysis and subsequent validation experiments revealed the anti-ferroptosis effect of CLY depends on the inhibition of ACSL4 acetylation in keratinocytes. Furthermore, ACSS2 was identified as a key protein promoting ACSL4 acetylation, which was inhibiting by CLY. Finally, six major compounds in CLY were identified as active constituents targeting ACSS2.

**Conclusion:**

Our findings demonstrate CLY promotes DFU wound healing by inhibiting keratinocyte ferroptosis through suppressing ACSS2-mediated ACSL4 acetylation, highlighting its potential as a rational therapeutic approach for the management of DFU.

**Graphical Abstract:**

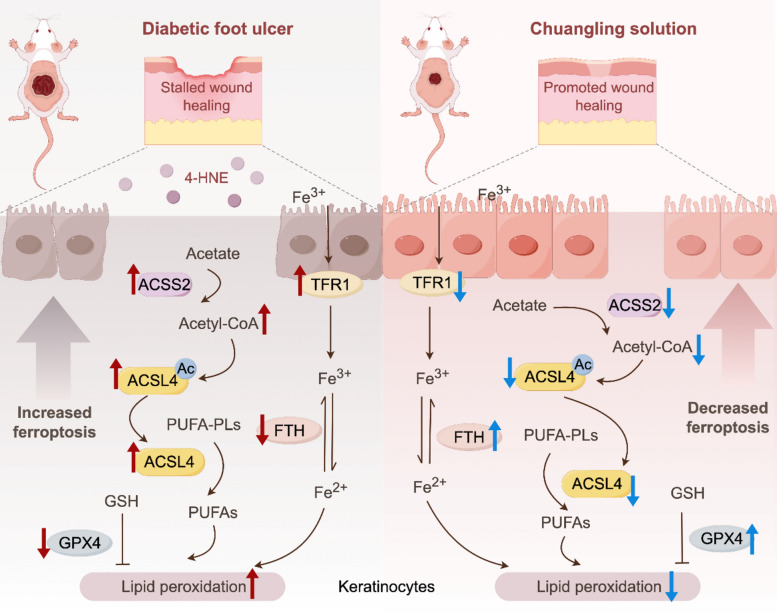

**Supplementary Information:**

The online version contains supplementary material available at 10.1186/s13020-026-01435-8.

## Introduction

Diabetic foot ulcers (DFU) are a common microvascular complication of diabetes, affecting approximately 19% to 34% of diabetic patients [24]. Persistent hyperglycemia and the accumulation of harmful metabolites contribute to chronic inflammation and vascular damage, thereby exacerbating wound infection and impairing healing [1]. Globally, DFU have emerged as a major cause of disability among diabetic patients, resulting in amputation rates as high as 20% and 5-year mortality rate of 30% approximately [2, 3]. However, current clinical strategies remain primarily focused on glycemic control and early intervention. In recent years, novel approaches such as drug-loaded wound dressings, cytokine therapy, skin flap transplantation, and negative pressure wound therapy have provided new avenues for DFU treatment [13, 28]. Nevertheless, high costs, inconsistent efficacy, and potential adverse effects continue to limit clinical benefits for patients. The need for effective pharmacological interventions for DFU remains unmet.

Numerous studies have reported that compound herbal formulations exhibit significant therapeutic efficacy in managing diabetic complications characterized by complex pathological mechanisms [19, 46]. Chuangling Ye (CLY) has been extensively utilized in clinical practice for treating various types of skin ulcers. CLY consists of Rhei Radix et Rhizoma (Dahuang), Carthami Flos (Honghua), Chebulae Fructus (Hezi), and Abelmoschi Corolla (Huangshukuihua), demonstrating remarkable efficacy in promoting the healing of ulcerative wounds. However, the underlying mechanisms of CLY against DFU remain incompletely understood.

Ferroptosis, an iron-dependent mode of cell death characterized predominantly by iron overload and lipid peroxidation, is implicated in the pathophysiology of various diabetic complications [6, 8, 20]. Clinical investigations have revealed significantly elevated levels of lipid peroxidation markers, such as malondialdehyde (MDA) and 4-hydroxynonenal (4-HNE), in the wound exudates of patients with DFUs, with their concentrations positively correlating with diabetes severity [12]. Furthermore, topical application of the ferroptosis inhibitor ferrostatin-1 (Fer-1) to wounds in diabetic rat models markedly suppressed the expression of oxidative stress and inflammation-related marker, thereby accelerating wound healing [17, 34]. Keratinocytes (KCs) play a pivotal role in wound healing, contributing to re-epithelialization through proliferation and migration, while simultaneously secreting cytokines that mitigate inflammatory responses and promote angiogenesis [21, 27]. Functional impairment of KCs under diabetic ulcer conditions contributes to the development of chronic non-healing wounds [21, 30]. Acyl-CoA synthetase long-chain family member 4 (ACSL4) is a key protein driving ferroptosis by promoting lipid peroxidation [5]. Previous studies have demonstrated that high glucose conditions induce the upregulation of ACSL4 expression in keratinocytes, facilitating iron deposition and lipid peroxidation, ultimately leading to cellular damage. And this detrimental effect can be reversed by ferroptosis inhibitors [40, 41]. Consequently, inhibiting ACSL4-mediated ferroptosis in keratinocytes represents a promising therapeutic intervention strategy for diabetic foot ulcers.

Acetylation of non-histone proteins represents a crucial post-translational modification that regulates protein function and stability [29]. Studies indicate that acetylation of ACSL4 at lysine 383 inhibits its ubiquitin-mediated degradation, thus enhancing protein stability and promoting cellular ferroptosis [45]. The acetylation levels of proteins are primarily regulated by the availability of the acetyl group donor acetyl-CoA, acetyltransferases (KATs), and deacetylases (HDACs and Sirtuins) [29]. Under physiological conditions, the primary sources of acetyl-CoA are the catabolism of glucose, fatty acids, and amino acids [37]. However, in metabolic diseases and diabetic complications, acyl-CoA synthetases short chain family member 2 (ACSS2)-mediated acetyl-CoA synthesis and subsequent acetylation modifications have received considerable attention [7, 18, 23]. Current research has identified upregulated ACSS2 expression in the renal tubules of diabetic patients and animal models, as well as in renal tubular epithelial cells stimulated by high glucose. Pharmacological inhibition or knockdown of ACSS2 has been shown to reduce protein acetylation levels and ameliorate renal function in diabetic animal models [22, 23]. Nevertheless, the role of ACSS2, a key enzyme regulating protein acetylation in dysregulated metabolic states, remains unclear in diabetic wound healing, and its regulatory function in ferroptosis has been rarely reported.

In view of the established clinical efficacy of CLY in accelerating wound healing, this study aimed to evaluate its therapeutic effects on DFUs and elucidate its underlying pharmacological mechanisms. We first examined the impact of CLY on wound healing rates and relevant biochemical markers in streptozotocin (STZ)-induced diabetic rats. Subsequently, leveraging the chemical constituents of CLY identified through UPLC-MS/MS analysis, we employed network pharmacology to predict potential mechanisms underlying its efficacy against DFU. This approach highlighted its potential regulatory role in ACSL4-mediated ferroptosis. We then utilized transcriptomics in an advanced glycation end products (AGEs)-induced HaCaT cell model to analyze the targets through which CLY regulates ferroptosis. This analysis identified that inhibiting ACSS2-mediated acetylation of ACSL4 might be a key mechanism of action. Finally, we tested the hypothesis that CLY inhibits ferroptosis in keratinocytes under DFU conditions by downregulating ACSS2-mediated ACSL4 acetylation through comprehensive validation in both in vitro and in vivo experiments.

## Material and methods

### Drugs

A mixture of *Rheum palmatum* L., *Terminalia chebula* Retz., *Carthamus tinctorius* L., and *Abelmoschus manihot* (L.) Medicus was soaked overnight and then refluxed three times (3 h, 2 h, and 1 h) using a solid-to-liquid ratio of 1:10. The combined filtrates were concentrated and made up to 250 mL with purified water to yield the CLY. To identify the chemical components, the extract was analyzed using UPLC-MS/MS. Recombinant bovine basic fibroblast growth factor (rb-bFGF, lot NO. 01241105) was purchased from Zhuhai Essex Bio-Pharmaceutical Co., Ltd.

### Animals and treatment

Male SD rats (8 weeks old) were purchased from Sparfo (Suzhou) Biotechnology Co., Ltd. (Suzhou, China) [SCXK (Su) 2022–0006]. All rats were maintained under constant temperature and humidity with access to food and water ad libitum. All experiments involving animals were performed following the protocol approved by the Animal Ethics Committee in Affiliated Hospital of Nanjing University of Traditional Chinese Medicine [2023DW-010-01]. One week after adaptive feeding, rats fed with high-fat diet for 4 weeks were received an intraperitoneal injection of 65 mg/kg of STZ, which controlled with age-matched rats fed with chow diet. Rats with random blood glucose levels greater than 16.7 mmol/L were used for subsequent experiments. A full-thickness skin wound with a diameter of 16 mm was created on the dorsal region of each diabetic rat. The entire skin and subcutaneous connective tissue within the marked region were completely excised and bluntly dissected until the deep fascia was exposed. Diabetic rats with wound were randomly divided into four groups: Model group, high dose of CLY group (CL–H, 0.45 g/mL), low dose of CLY group (CL-L, 0.225 g/mL), and positive control rb-bFGF group (3.6 μg/mL), the dressings were changed once a day. The low dose (CL-L) represents the concentration used in clinical practice, whereas the high dose (CL–H) is the concentration previously demonstrated to exert significant efficacy in promoting skin wound healing in diabetic animals in our published study [9]. On days 3, 7, and 12 post-treatment, wound images were captured including a scale bar within the field of view.

### Histological analysis

Regenerated skin samples were collected on day 21 from sacrificed rats. The samples were fixed using 4% paraformaldehyde (PFA), embedded in paraffin, and sectioned for H&E and Masson staining. Immunohistochemical staining for vascular endothelial growth factor (VEGF, Bioss, Beijing, China) was conducted to assess angiogenesis.

### Multiplex immunochemical staining

For 4 μm skin sections, antigen retrieval was performed by heating in citrate buffer after deparaffinization and rehydration. Subsequently, the sections were treated with 3% hydrogen peroxide (Sigma-Aldrich, USA) for 30 min and blocked with immunostaining blocking solution (P0260, Beyotime, China) for 30 min. The sections were sequentially incubated with primary antibody, secondary antibody, fluorophore (TSA520 or TSA570), and DAPI. After completing the first round of protein labeling, the antibodies were dissociated by heating in citrate buffer, and the staining procedure was repeated with the corresponding antibodies for subsequent targets. All fluorophores and secondary antibodies were provided in the mIHC kit (Absin Bioscience, Shanghai, China). The antibodies and their dilution ratios were as follows: anti-GPX4 (1:500, ab125066, Abcam, USA), anti- KRT14 (1:500, 60320-1-Ig, Proteintech, China).

### Chemical component identification

Chemical component identification was performed using a UPLC system coupled to a Q-Exactive Plus mass spectrometer (Thermo Scientific). Separation was performed on an ACQUITY UPLC HSS T3 column (2.1 × 100 mm, 1.8 µm, Waters) at 40 °C with a flow rate of 0.3 mL/min. The mobile phase consisted of (A) 0.1% formic acid in water and (B) 0.1% formic acid in acetonitrile. A gradient elution was applied as follows: 0–6 min, 0–48% B; 6–10 min, 48–100% B; 10–12 min, 100% B; 12–15 min, 100–0% B. The injection volume was 6 µL. Mass spectrometry was operated in both positive and negative ESI modes. Settings included spray voltages of 3.8 kV (+) and 3.2 kV (−), capillary temperature of 320 °C, and sheath gas flow of 30 arb. Full-scan MS spectra (m/z 75–1050) were acquired at a resolution of 70,000, followed by data-dependent MS^2^ scans of the top 10 ions using stepped collision energies. Raw data were processed with MS-DIAL (v.5.1230912) for peak alignment and feature extraction. Chemical components in CL were identified by accurate mass matching (< 10 ppm) and MS/MS spectral fragment ion comparison (< 20 ppm).

### Network pharmacology analysis

The Traditional Chinese Medicine Systems Pharmacology (TCMSP) database was utilized to predict potential targets of CL solution, with screening criteria defined as oral bioavailability (OB) ≥ 30% and drug-likeness (DL) ≥ 0.18. The GeneCards and OMIM databases were employed to identify disease-related targets using the keywords “Diabetic wound” and “Diabetic foot ulcers”. The intersecting genes between CL solution and disease were analyzed and visualized using Cytoscape software (version 3.10.2). Functional enrichment analyses, including Gene Ontology (GO) and Kyoto Encyclopedia of Genes and Genomes (KEGG) pathway enrichment, were performed in DAVID database.

### Measurement of lipid peroxidation and iron levels

Malondialdehyde (MDA, Solarbio, Beijing, China), 4-Hydroxynonenal (4-HNE, Solarbio, Beijing, China), Glutathione (GSH, Solarbio, Beijing, China), Superoxide dismutase (SOD, Solarbio, Beijing, China) activity, and ferrous ion content (Solarbio, Beijing, China) in tissues or cells were measured using commercial assay kits, according to the manufacturer’s instructions.

### Western blot

Skin tissues and cells were collected and lysed in RIPA lysis buffer (Beyotime, Shanghai, China) supplemented with a protease inhibitor. Protein samples were separated by SDS-PAGE and subsequently transferred onto 0.22 μm PVDF membranes. After blocking with 5% bovine serum albumin (Bioss, Beijing, China) for 2 h, the membranes were incubated overnight with primary antibodies, including TFR1 (Bioss, Beijing, China), ACSL4 (Proteintech, Wuhan, China), ac-ACSL4 (ABclonal, Wuhan, China), GPX4 (Proteintech, Wuhan, China), FTH (Wanlei, Shenyang, China), ACSS2 (Proteintech, Wuhan, China) and GAPDH (Wanlei, Shenyang, China). The membranes were then incubated with HRP-conjugated secondary antibody (Wanlei, Shenyang, China) for 2 h and achieved using ECL chemiluminescence (Wanlei, Shenyang, China).

### Preparation of advanced glycation end products (AGE)

Bovine albumin (Servicebio, Wuhan, China) was mixed with 40% methylglyoxal (Macklin, Shanghai, China) and dissolved in phosphate buffer to obtain a 10 mg/mL AGE solution and incubated at 37 °C in the dark for 14 days. The incubated solutions were transferred into pre-activated dialysis membranes and dialyzed against 10 mmol/L phosphate buffer for 7 days to remove unbound methylglyoxal. The fluorescence intensity of AGE was measured at 370 nm excitation and 440 nm emission for semi-quantitative analysis of AGE content.

### Cell culture and treatment

The human keratinocytes cells (HaCaT cells, ATCC) were cultured in DMEM medium (KeyGEN, Nanjing, China) supplemented with 10% fetal bovine serum (Gibco, USA) at 37 ℃, 5% CO_2_. HaCaT cells were induced with 400 μg/mL AGE for 24 h to establish in vitro model while 100 and 200 μg/mL CL solution were used for cell experiments. In the acetyl‑CoA rescue experiment, cells were treated with 1 mM acetyl‑CoA (MCE, Shanghai, China) simultaneously with AGE and CL solution incubation.

### Measurement of ROS

Intracellular ROS levels were detected using the fluorescent probe DCFH-DA (Beyotime, Shanghai, China). HaCaT cells after treatment were incubated with 10 μM DCFH-DA at 37 °C for 30 min in the dark. After incubation, the cells were washed three times with PBS to remove excess probe. Fluorescence was measured with a microplate reader at an excitation wavelength of 488 nm and an emission wavelength of 525 nm.

### Transmission electron microscopy (TEM)

HaCaT cells were fixed with 2.5% glutaraldehyde in phosphate buffer (Servicebio, Wuhan, China) at 4 °C overnight. After dehydration through ethanol, the cells were embedded in epoxy resin and stained with uranyl acetate and lead citrate. Images were acquired using a transmission electron microscope (HT7800, Hitachi) operating at 80 kV.

### RNA-sequence

The RNA of AGE-induced HaCaT cells with or without CL treatment were extracted by total RNA extraction reagent (Vazyme, Nanjing, China). The differentially expressed genes (DEGs) were identified through *P* < 0.05 and log_2_FoldChange > 1 or < 1. Functional enrichment analysis of DEGs was performed using the clusterProfiler package (v4.0) in R for GO and KEGG enrichment analysis.

### Real-time PCR (RT-qPCR)

Total RNA was extracted using RNA extraction reagent and reverse-transcribed into cDNA with All-in-One First-Strand Synthesis Master Mix (Best Enzymes, Lianyungang, China). qPCR was performed using Taq-HS SYBR Green qPCR Premix (Best Enzymes, Lianyungang, China) on a QuantStudio 6 Flex system. The 2^(^−ΔΔ^Ct) method was applied to calculate relative gene expression levels.

### Measurement of acetyl-CoA levels

Acetyl-CoA levels were measured by Acetyl Coenzyme A ELISA Kit (Elabscience Biotechnology Co., Ltd.) following manufacturer’s instructions.

### Transfection of plasmid

HaCaT cells were seeded in plates and cultured until they reached 70–80% confluence. Plasmid DNA (KeyGEN BioTECH, Nanjing, China) or siRNA targeting SIRT1 (forward: 5'-GUGGCAGAUUGUUAUUAAUTT-3'; reverse: 5' AUUAAUAACAAUCUGCCACTT 3'), and Lipofectamine 3000 transfection reagent (Thermo Fisher Scientific, USA) were separately diluted in Opti-MEM medium (Gibco, USA), gently mixed, and incubated at room temperature for 15 min to allow complex formation. The resulting mixture was then added dropwise to the cells, and the medium was replaced with fresh medium 6 h post-transfection.

### Statistics analysis

The Kolmogorov–Smirnov test was used to assess the normality of the data. Multiple group comparisons were analyzed using one-way ANOVA followed by Tukey’s tests with GraphPad Prism software (version 9). All data are expressed as Mean ± SEM. Statistical significance was defined as *P* < 0.05.

## Results

### CLY accelerates diabetic wound healing and angiogenesis

**Fig. 1:**
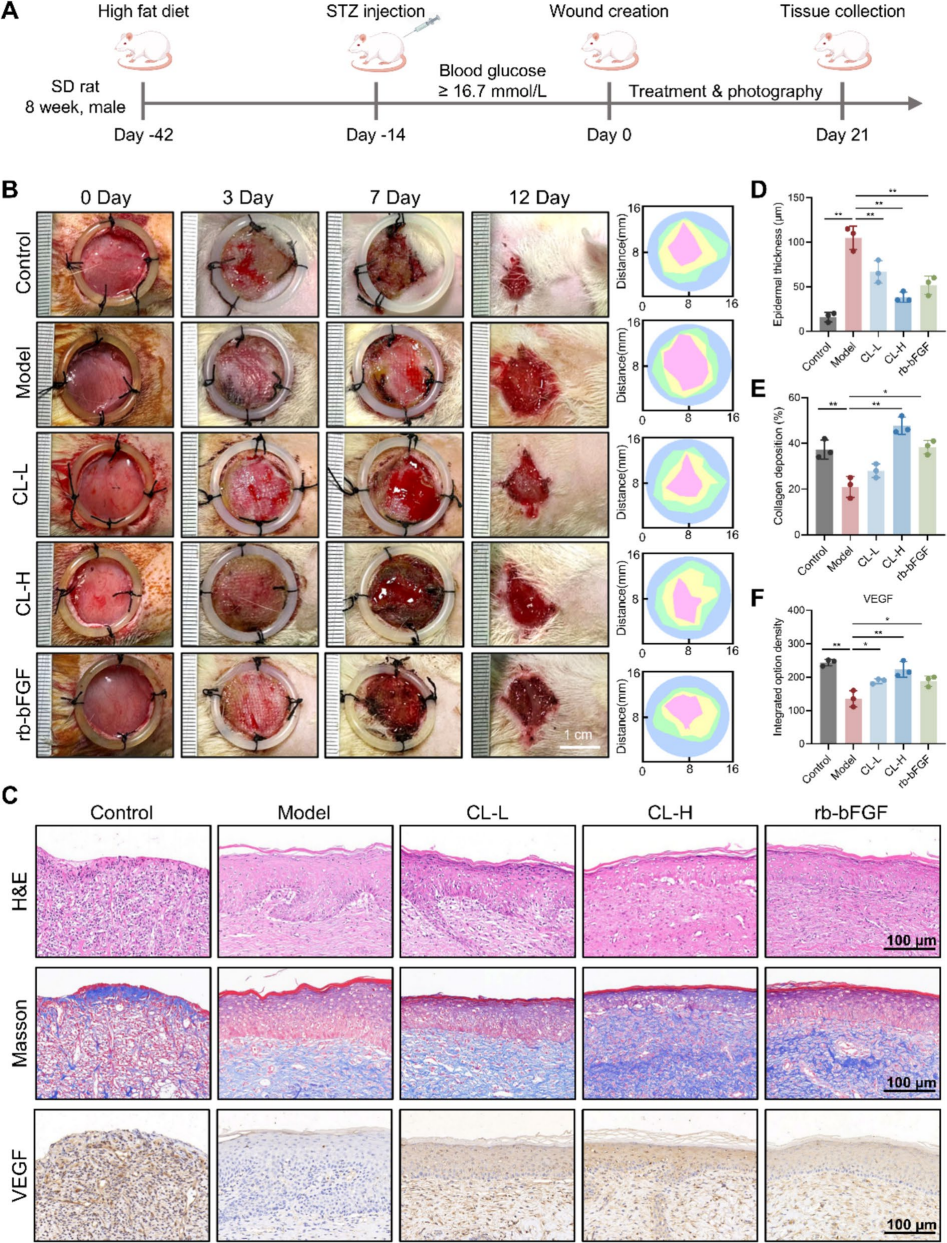
CLY accelerates diabetic wound healing and angiogenesis. **A** Schematic diagram of the animal experimental procedure. **B** Representative images of rat skin wounds at different time points post-repair and the corresponding trace of wound-bed closure. Blue, day 0; green, day 3; yellow, day 7; pink day 12. **C** Histological sections of rat wound skin tissue stained with H&E, Masson's trichrome, and VEGF immunohistochemistry. **D**–**F** Quantitative analysis of **D** neo-epidermal thickness, **E** collagen deposition level, and **F** VEGF expression level in rat wound skin, *n* = 3 per group. Data are presented as mean ± SEM. *P < 0.05, **P < 0.01

DFU rats were administrated with CLY or rb-bFGF for 21 days to evaluate its therapeutic effects (Fig. 1A). As illustrated in Fig. 1B, DFU rats exhibited a slower wound healing rate compared with the control group. However, treatment with CLY significantly accelerated the healing process, as evidenced by the reduced wound area. Additionally, histological staining was performed to further validate the therapeutic efficacy of CLY (Fig. 1C). H&E staining indicated a significant increase in epidermal thickness in DFU rats, which was effectively attenuated following administration of CLY (Fig. 1D). Masson staining revealed that CLY significantly increased the collagen deposition rate in DFU rats compared with that in the model group (Fig. 1E). Notably, IHC staining suggested that CLY inhibited the DFU-induced downregulation of VEGF expression (Fig. 1F). Collectively, our findings demonstrate that CLY accelerates diabetic wound healing and promotes angiogenesis, thereby exerting a potent therapeutic effect on DFU.

### Network pharmacology analysis of CLY in treating DFU

**Fig. 2:**
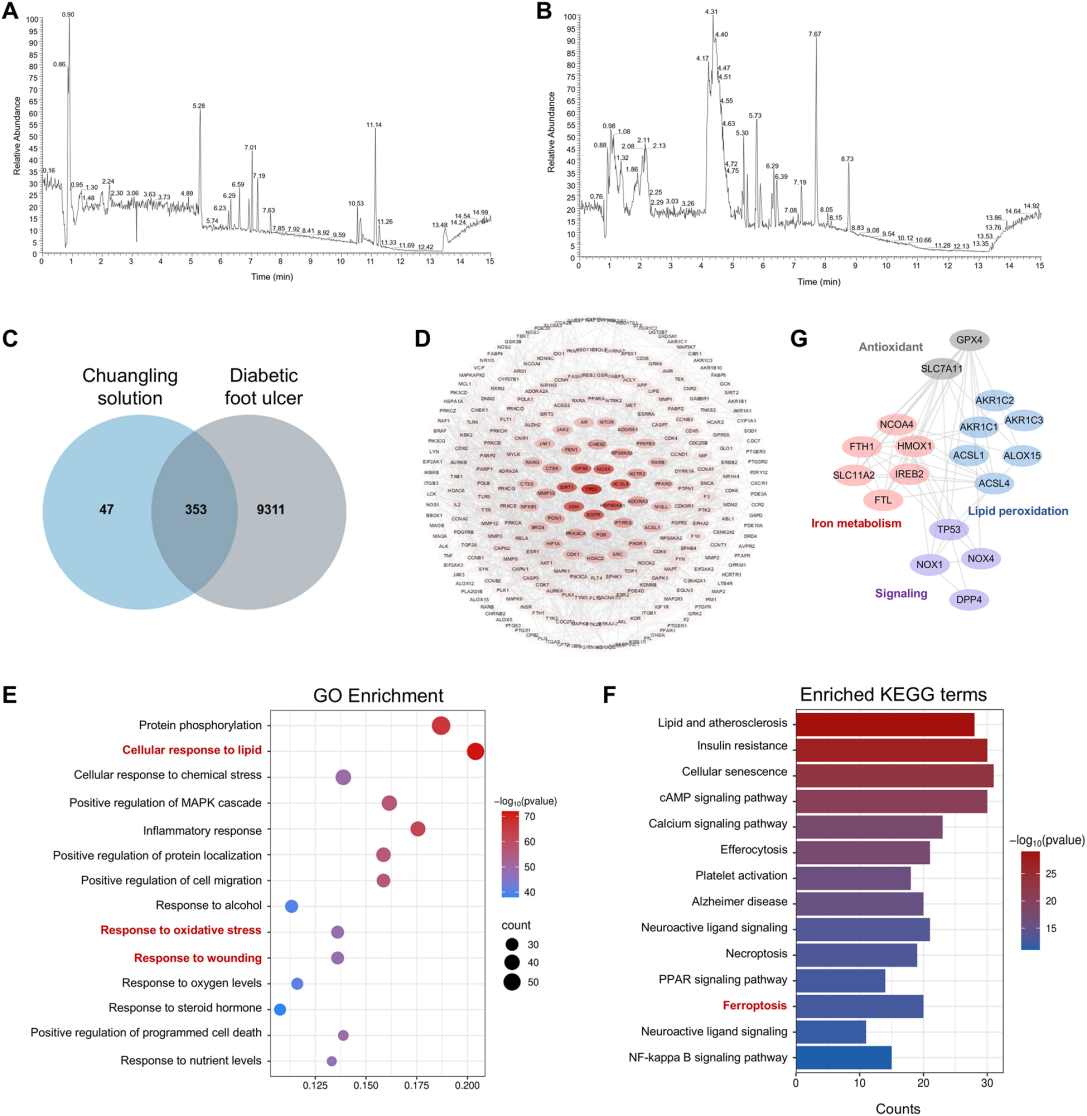
Network pharmacology analysis of CLY in treating DFU. **A**, **B** Total ion chromatograms (TIC) of Chuangling Ye detection by LC–MS in the **A** positive and **B** negative ion modes. **C** Venn diagram showing the intersection of predicted compound targets from CLY and known therapeutic targets for diabetic foot ulcer (DFU). **D** PPI network of the overlapping targets identified in panel **C**. **E**–**F** Functional enrichment analysis of the overlapping targets from panel **C**: **E** bubble plot of GO enrichment analysis and **F** bar plot of KEGG pathway enrichment analysis. **G** PPI network diagram of the targets in the biological processes and pathways identified in functional enrichment analysis. *P < 0.05, **P < 0.01

To further elucidate the mechanism underlying CLY accelerating diabetic wound healing, a network pharmacology analysis was performed. As shown in Fig. 2A, B, the chemical components of CLY were identified through metabolomics analysis, and the total ion flow profiles in both negative and positive ion modes were present. Additionally, the top 20 most abundant chemical components were listed in Table 1. A total of 353 potential targets for CLY in treating DFU were predicted using the TCMSP, OMIM, and GeneCards databases (Fig. 2C). The PPI network of all predicted targets was established using STRING databases and exhibited in Fig. 2D. Next, GO enrichment analysis and KEGG enrichment analysis were conducted based on these 353 potential targets. As shown in Fig. 2E, biological processes such as “Cellular response to lipid”, “Response to wounding”, and “Response to oxidative stress” were significantly enriched through GO analysis. KEGG enrichment analysis revealed that the “Ferroptosis” pathway was enriched (Fig. 2F). Furthermore, the PPI network of core ferroptosis-related potential targets was visualized using Cytoscape software. A total of 18 core ferroptosis-related potential targets, including GPX4, ACSL4, and FTH1, were identified and categorized into four functional classes: “Antioxidant”, “Iron metabolism”, “Lipid peroxidation”, and “Signaling” (Fig. 2G). In short, these findings suggest that CLY ameliorates DFU is associated with regulating ferroptosis.

**Table 1:** LC–MS/MS data of characterized compounds in CLY (top 20)

Number	Name	Formula	RT (min)	m/z	Class
1	Gallic acid	C_7_H_6_O_5_	4.34	169.013	Benzene and substituted derivatives
2	Bergapten	C_12_H_8_O_4_	0.88	215.032	Coumarins and derivatives
3	Ellagic acid	C_14_H_6_O_8_	6.29	300.999	Tannins
4	Gallotannin	C_27_H_24_O_18_	5.87	635.09	Tannins
5	Gastrodin	C_13_H_18_O_7_	7.21	309.087	Organooxygen compounds
6	Isoquercitrin	C_21_H_20_O_12_	6.23	465.103	Flavonoids
7	Rhein	C_15_H_8_O_6_	8.05	283.025	Anthracenes
8	Quercetin	C_15_H_10_O_7_	7.09	301.035	Flavonoids
9	Pyrogallol	C_6_H_6_O_3_	5.40	127.039	Phenols
10	Genistin	C_21_H_20_O_10_	6.92	431.097	Isoflavonoids
11	Liquiritin	C_21_H_22_O_9_	6.32	419.134	Flavonoids
12	Kaempferol	C_15_H_10_O_6_	7.46	285.04	Flavonoids
13	Perlolyrine	C_16_H_12_N_2_O_2_	6.41	265.097	Harmala alkaloids
14	Gossypetin	C_15_H_10_O_8_	6.71	317.03	Flavonoids
15	Eudesmin	C_22_H_26_O_6_	8.07	387.18	Furanoid lignans
16	Rubiadin	C_15_H_10_O_4_	6.98	253.05	Anthracenes
17	1,2,3,6-Tetragalloylglucose	C_34_H_28_O_22_	6.14	787.1	Tannins
18	Quercetagitrin	C_21_H_20_O_13_	6.02	481.098	Flavonoids
19	trans-Aconitic acid	C_6_H_6_O_6_	0.98	173.008	Carboxylic acids and derivatives
20	Astragalin	C_21_H_20_O_11_	6.43	449.108	Flavonoids

### CLY inhibits ferroptosis and lipid peroxidation in DFU rats

**Fig. 3:**
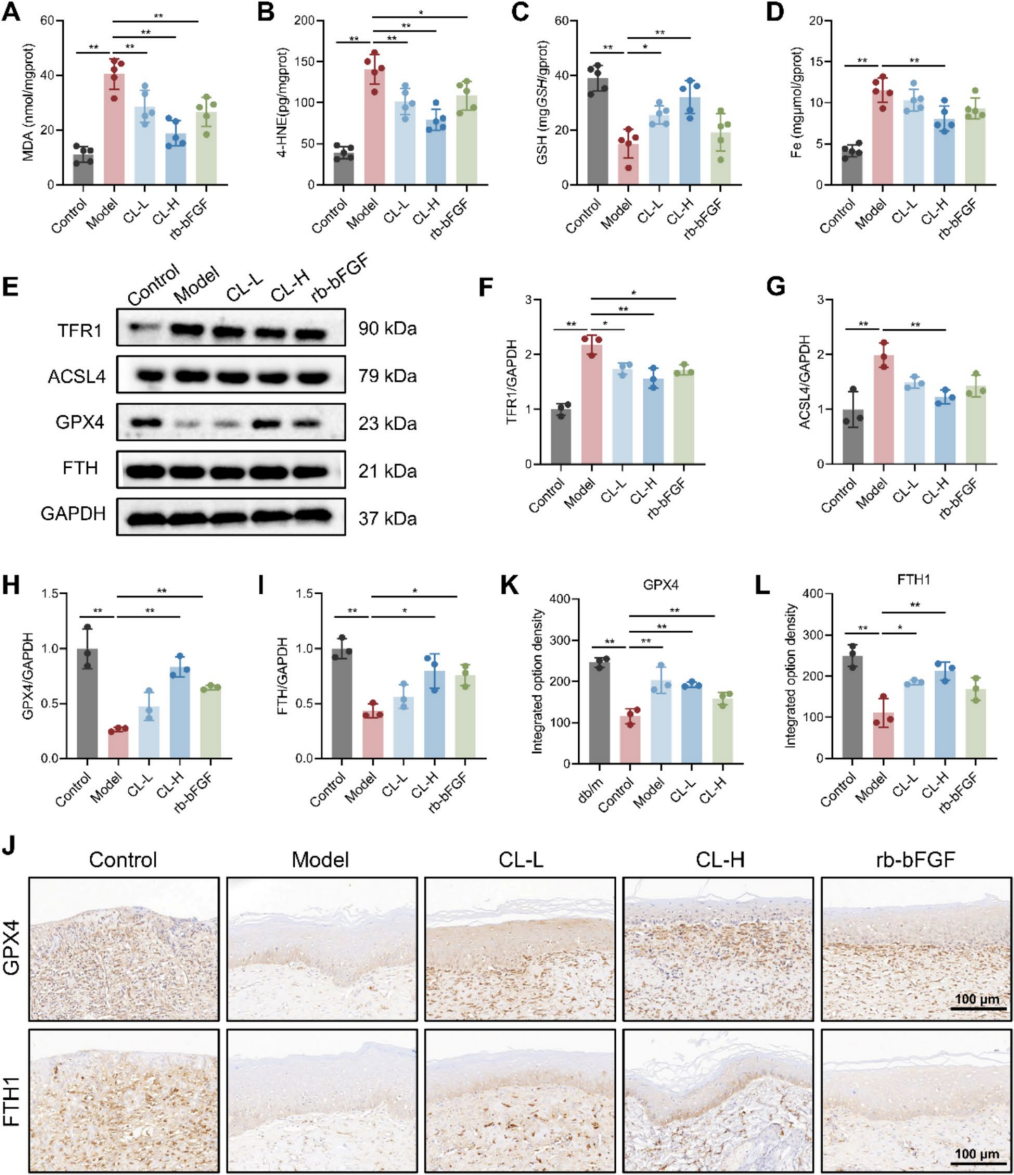
CLY inhibits ferroptosis and lipid peroxidation in DFU rats. **A**–**D** The levels of **A** MDA, **B** 4-HNE, **C** GSH, and **D** iron in rat wound skin tissue, *n* = 5 per group. **E** Representative Western blot bands and quantitative analysis of the protein expression levels of **F** TFR1, **G** ACSL4, **H** GPX4, and **I** FTH, *n* = 3 per group. **J** Representative immunohistochemical staining images of rat wound skin tissue sections and quantitative analysis of the positive expression area for **K** GPX4 and **L** FTH1, *n* = 3 per group. Data are presented as mean ± SEM. *P < 0.05, **P < 0.01

Lipid peroxidation and dysregulation of iron metabolism are hallmark features of ferroptosis, both of which were measured in this study. As shown in Fig. 3A, B, DFU rats exhibited significantly increased levels of MDA and 4-HNE in skin tissue compared to the control group, whereas treatment with CLY or rb-bFGF effectively attenuated these increases. Similarly, CLY significantly restored GSH levels and reduced elevated iron content in DFU rats (Fig. 3C, D). Subsequently, the expression of ferroptosis-related proteins was assessed using Western blot analysis and IHC staining. As illustrated in Fig. 3E–I, CLY treatment led to a significant decrease in the expression of TFR1 and ACSL4, along with increased expression of GPX4 and FTH1, compared to the model group. Moreover, IHC staining results further validated the regulatory effects of CLY on ferroptosis-related proteins, as indicated by elevated expression of GPX4 and FTH1 (Fig. 3K-J). Collectively, these findings demonstrate that CLY effectively suppresses ferroptosis and lipid peroxidation in DFU rats.

### CLY accelerates diabetic wound healing through inhibiting ferroptosis

**Fig. 4:**
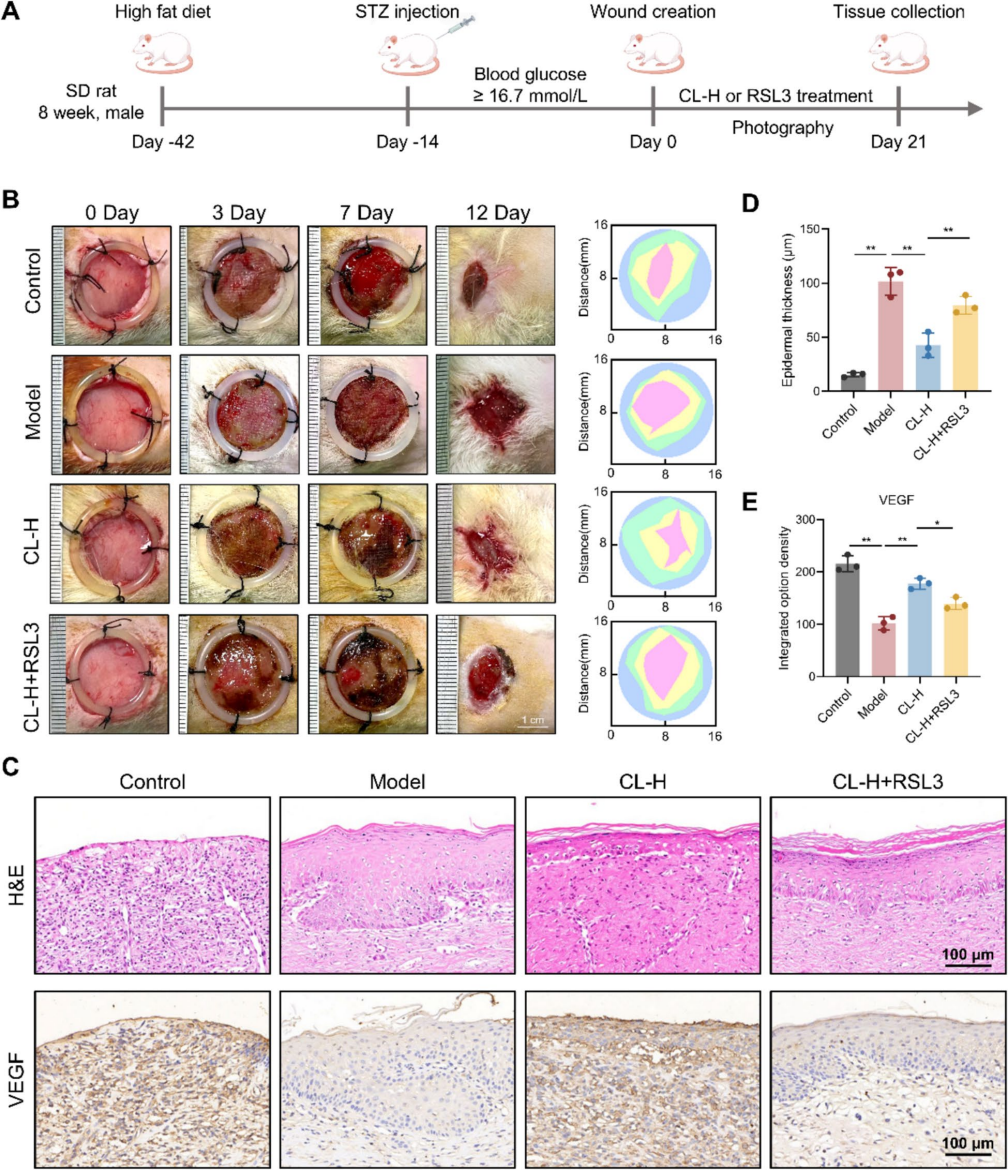
CLY accelerates diabetic wound healing through inhibiting ferroptosis. **A** Schematic diagram of the animal experimental procedure. **B** Representative images of rat skin wounds at different time points post-repair and the corresponding trace of wound-bed closure. Blue, day 0; green, day 3; yellow, day 7; pink day 12. **C** Histological sections of rat wound skin tissue stained with H&E and VEGF immunohistochemistry. **D**–**E** Quantitative analysis of **D** neo-epidermal thickness and **F** VEGF expression level in rat wound skin, *n* = 3 per group. Data are presented as mean ± SEM. *P < 0.05, **P < 0.01

Next, ferroptosis inducer RSL3 was employed to further validate the mechanism underlying the beneficial effects of CLY on DFU (Fig. 4A). As illustrated in Fig. 4B, DFU rats treated with CLY exhibited a significantly accelerated wound healing rate compared to those in the Model group. However, co-treatment with CLY and RSL3 markedly impaired wound closure, as indicated by a larger residual wound area. Additionally, HE staining indicated that RSL3 counteracted the therapeutic effect of CLY on epidermal regeneration, manifested as a significant increased epidermal thickness (Fig. 4C, D). Consistent with these morphological changes, IHC staining showed that RSL3 treatment substantially downregulated the expression of VEGF, which had been increased by CLY (Fig. 4C, E). In short, above findings demonstrate that CLY promotes diabetic wound healing primarily through inhibiting ferroptosis.

### CLY reduces ferroptosis in AGEs-induce HaCaT cells

**Fig. 5:**
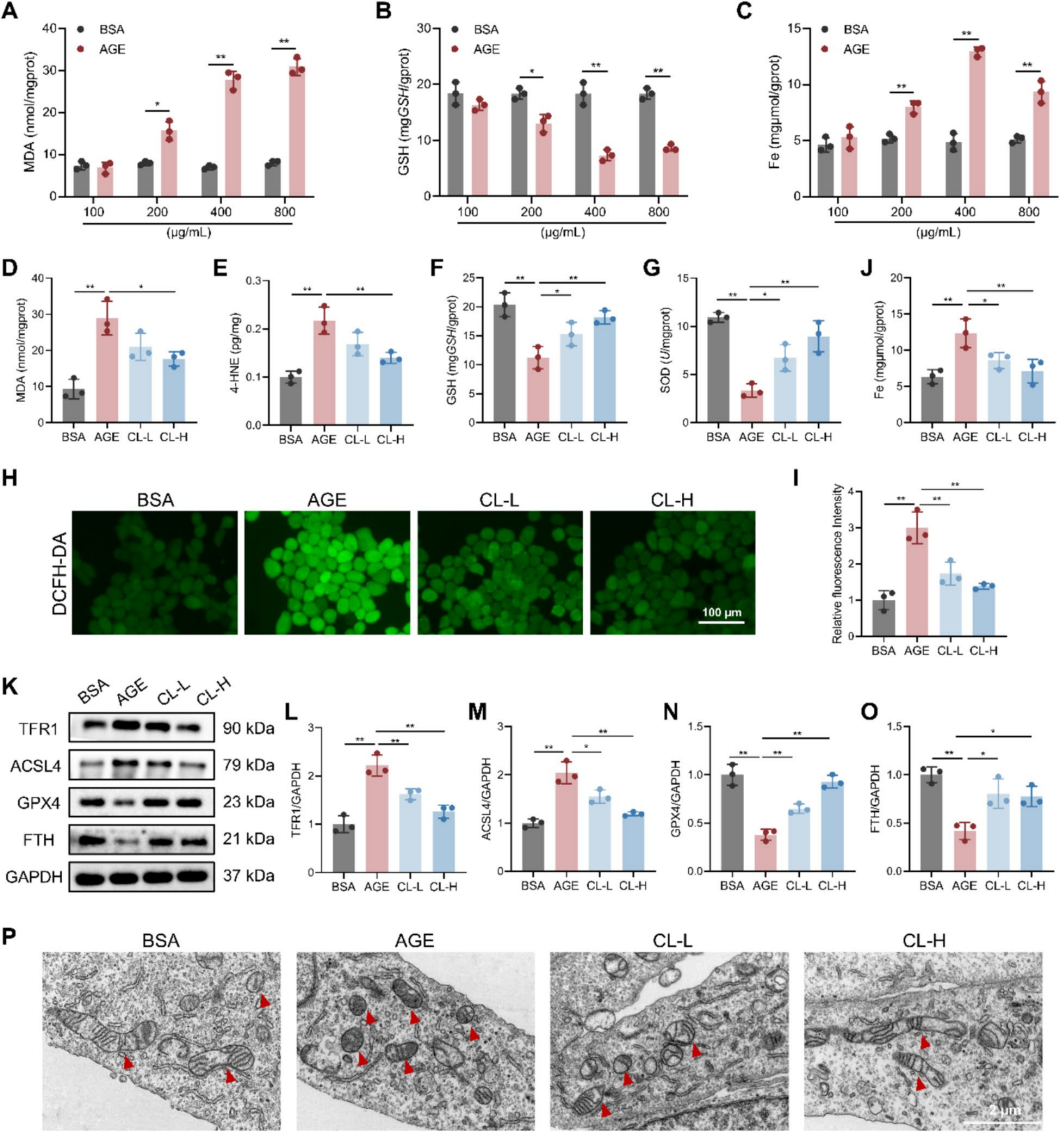
CLY reduces ferroptosis in AGEs-induce HaCaT cells. **A**–**C** Effects of different concentrations of AGEs on the levels of **A** MDA, **B** GSH, and **C** iron in HaCaT cells, *n* = 3 per group. **D**–**J** Effects of CLY on AGEs-induced changes in **D** MDA, **E** 4-HNE, **F** GSH, **G** iron content, and **J** SOD activity in HaCaT cells, *n* = 3 per group. **H** Representative images of ROS staining using DCFH-DA and **I** quantitative analysis of fluorescence intensity, *n* = 3 per group. **K** Representative western blot bands and quantitative analysis of protein expression levels of **L** TFR1, **M** ACSL4, **N** GPX4, and **O** FTH in HaCaT cells, *n* = 3 per group. **P** Representative transmission electron microscopy images of subcellular structures; red arrows indicate mitochondria. Data are presented as mean ± SEM. *P < 0.05, **P < 0.01

High glucose-induced ferroptosis in keratinocytes has been reported to accelerate the pathological progression of DFU [45]. Immunofluorescence staining results also showed that GPX4 expression in Keratin14-positive keratinocytes of diabetic rat wound skin was significantly decreased, while CLY treatment significantly restored GPX4 levels in keratinocytes (Fig. S1). Thus, the anti-ferroptotic effect of CLY on keratinocytes warrants further investigation. In vitro, HaCaT cells were incubated with various concentrations of AGEs (100, 200, 400, and 800 μg/mL), with BSA at the same concentrations used as controls. As shown in Fig. 5A-C, 400 μg/mL AGEs significantly increased MDA, GSH, and iron levels; therefore, this concentration was selected for subsequent experiments. Following treatment with CLY, AGEs-induced HaCaT cells exhibited a significant reduction in MDA, 4-HNE and iron levels (Fig. 5D-E, J). Similarly, a significant increase in GSH and SOD levels was observed in CL solution-treated groups compared to the AGE group (Fig. 5F-G). Next, ROS levels were detected using the DCFH-DA fluorescent probe, and the results indicated that AGEs induced a significant elevation in ROS levels in HaCaT cells. However, CLY effectively suppressed this increase (Fig. 5H-I). Additionally, the expression of ferroptosis-related proteins was assessed by Western blot analysis. As illustrated in Fig. 5K-O, CLY treatment led to a significant decrease in the expression of TFR1 and ACSL4, along with an increase in the expression of GPX4 and FTH1, compared to the AGE group. Mitochondrial health is a key indicator for evaluating ferroptosis. Here, AGE caused a reduction in mitochondrial volume and loss of mitochondrial cristae in HaCaT cells, whereas CLY treatment significantly reversed these alterations (Fig. 5P). In conclusion, these findings indicate that CLY reduces ferroptosis in AGEs-induce HaCaT cells.

### CLY suppresses ferroptosis through reducing ACSL4 protein stability

**Fig. 6:**
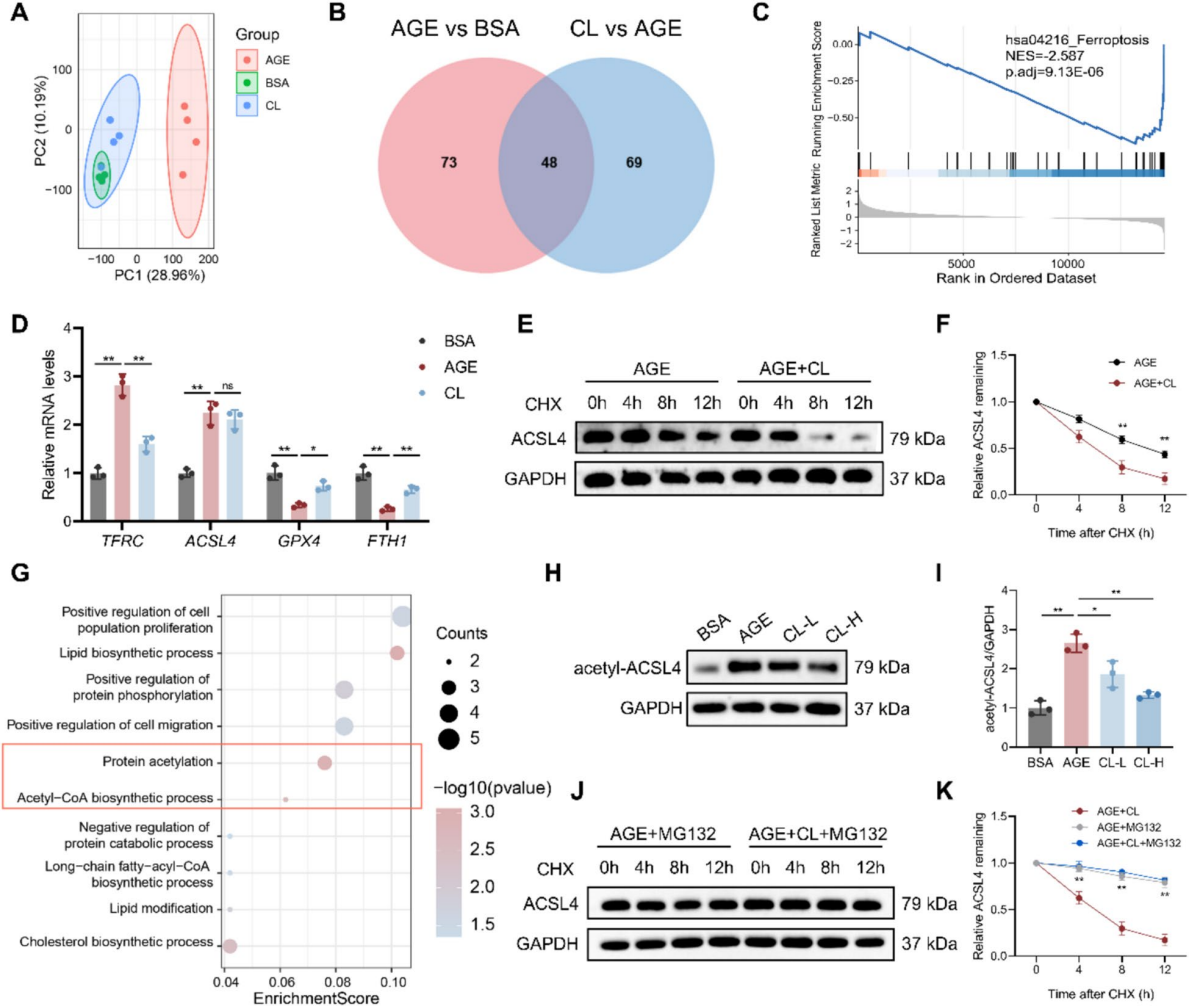
CLY suppresses ferroptosis through reducing ACSL4 protein stability. **A**, **B** **A** Principal component analysis (PCA) plot, and **B** Venn diagram showing the number of differentially expressed genes (DEGs) of RNA-seq data from HaCaT cells treated with AGEs or CLY. **C** GSEA plot demonstrating the enrichment of the ferroptosis pathway in CLY-treated vs. AGE-treated HaCaT cells. **D** mRNA expression levels of ferroptosis-related genes in HaCaT cells, *n* = 3 per group. **E** Representative western blot bands and **F** corresponding quantitative analysis of ACSL4 protein expression in HaCaT cells treated with AGEs or CLY following cycloheximide (CHX) chase, *n* = 3 per group. **G** GO enrichment analysis bubble plot for the overlapping DEGs from in the Venn diagram (**B**). **H** Representative western blot bands and **I** quantitative analysis of acetylated ACSL4 protein levels in HaCaT cells, *n* = 3 per group. **J** Representative western blot bands and **K** quantitative analysis of ACSL4 protein expression in HaCaT cells treated with the proteasome inhibitor MG132 and CLY following CHX chase, *n* = 3 per group. Data are presented as mean ± SEM. *P < 0.05; **P < 0.01; ns, not significant

RNA-seq was performed on AGEs-induced HaCaT cells treated with CLY to further investigate the reason for reduced ferroptosis in keratinocytes during DFU. Principal component analysis revealed a clear distinction between the transcriptional profiles of AGEs-treated cells and the BSA-treated control group, while CLY intervention in AGEs-treated cells brought them closer to the control group (Fig. 6A). Further screening identified differentially expressed genes (DEGs) between the AGE and BSA groups, as well as between the CL and AGE groups. A total of 48 DEGs was identified and exhibited in Fig. 6B. GSEA results indicated that the “Ferroptosis” pathway was significantly inhibited following administration of CLY (Fig. 6C). RT-qPCR results showed that CLY effectively decreased the upregulation of *TFRC* levels and reversed the downregulation of *GPX4* and *FTH1* levels (Fig. 6D). Notably, although CLY had no significant effect on AGEs-induced *ACSL4* mRNA levels (Fig. 6D), it significantly inhibited ACSL4 protein expression. Subsequently, a cycloheximide (CHX) chase assay was conducted [45], revealing that CLY significantly decreased ACSL4 protein expression after 8 h of treatment compared to the AGE group, suggesting that CLY decreases ACSL4 protein stability (Fig. 6E-F). GO enrichment analysis of the intersecting genes revealed that CLY significantly regulates biological processes in keratinocytes, including lipid biosynthesis, cell migration, protein degradation, acetyl-CoA biosynthesis, and protein acetylation (Fig. 6G). Given previous report indicating that ACSL4 acetylation enhances its stability, thereby promoting ACSL4-mediated ferroptosis, we further examined the acetylation level of ACSL4. A significant decrease in acetyl-ACSL4 protein expression in AGEs-induced HaCaT cells was observed after treatment with CLY (Fig. 6H, I). As illustrated in Fig. 6J, K, administration of MG-132 significantly attenuated the CLY-induced degradation of ACSL4. Collectively, these findings demonstrate that CLY suppresses ferroptosis by reducing the protein stability of ACSL4.

### CLY inhibits ACSL4 expression associated with reducing acetyl-CoA production and acetylation modification

**Fig. 7:**
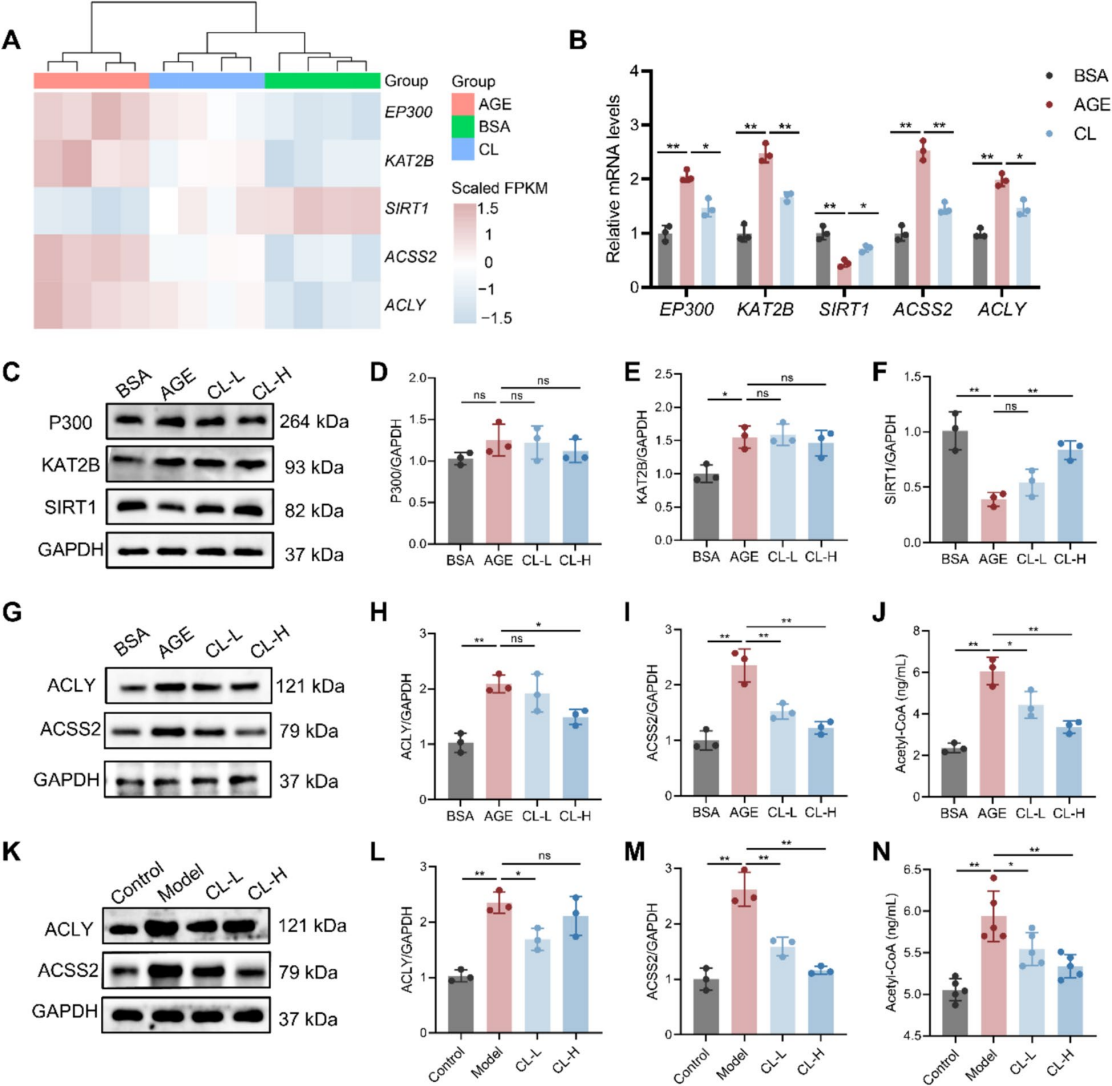
CLY inhibits ACSL4 expression associated with reducing acetyl-CoA production and acetylation modification. **A** Heatmap displaying RNA-seq results of genes significantly affected by AGEs and CLY treatments in protein acetylation and acetyl-CoA biosynthesis pathways. **B** RT-qPCR validation of selected genes from (A), *n* = 3 per group. **C** Representative western blot bands of protein acetylation-related proteins and quantitative analysis of protein expression levels for **D** P300, **E** KAT2B, and **F** SIRT1 in HaCaT cells, *n* = 3 per group. **G** Representative western blot bands of acetyl-CoA biosynthesis-related proteins and quantitative analysis of protein expression levels for **H** ACLY and **I** ACSS2 in HaCaT cells, *n* = 3 per group. **J** Intracellular acetyl-CoA content in HaCaT cells, *n* = 3 per group. **K** Representative western blot bands of proteins and quantitative analysis of protein expression levels in rat tissue for **L** ACLY and **M** ACSS2 in rat wound skin tissue, *n* = 3 per group. **N** Acetyl-CoA content in rat wound skin tissue, *n* = 5 per group. Data are presented as mean ± SEM. *P < 0.05; **P < 0.01; ns, not significant

As shown in Fig. 7A, a heatmap displayed significantly altered genes in the “acetyl-CoA biosynthesis” and “protein acetylation” gene sets of AGE- and CLY-treated HaCaT cells, suggesting that AGEs may affect cellular function by promoting acetyl-CoA production and protein acetylation modifications. RT-qPCR validation experiments revealed that CLY significantly attenuated AGEs-induced upregulation of *EP300*, *KAT2B*, *ACSS2*, and *ACLY*, while increasing *SIRT1* levels (Fig. 7B). Next, the expression levels of these acetylation-related protein were evaluated in vitro. No significant changes in P300 or KAT2B expression were observed following CLY treatment; however, SIRT1 levels were elevated, whereas ACSS2 and ACLY levels were reduce (Fig. 7C–I). Furthermore, CLY treatment decreased acetyl-CoA production compared to the AGE group (Fig. 7J). Similarly, DFU rats exhibited significantly upregulated ACSS2 and ACLY protein expression as well as increased acetyl-CoA production, both of which were suppressed by CLY treatment (Fig. 7K–N). In vitro, acetyl-CoA supplementation increased the levels of acetyl-CoA in cells (Fig. S3A), and counteracted the role of CLY in inhibiting acetylation of ACSL4 (Fig. S3B) and in alleviating oxidative stress (Fig. S3C) induced by AGEs in keratinocytes. Taken together, above data demonstrate that CLY inhibits ACSL4 acetylation is associated with reducing acetyl-CoA production.

### CLY inhibits ACSL4 acetylation through decreasing ACSS2 expression

**Fig. 8:**
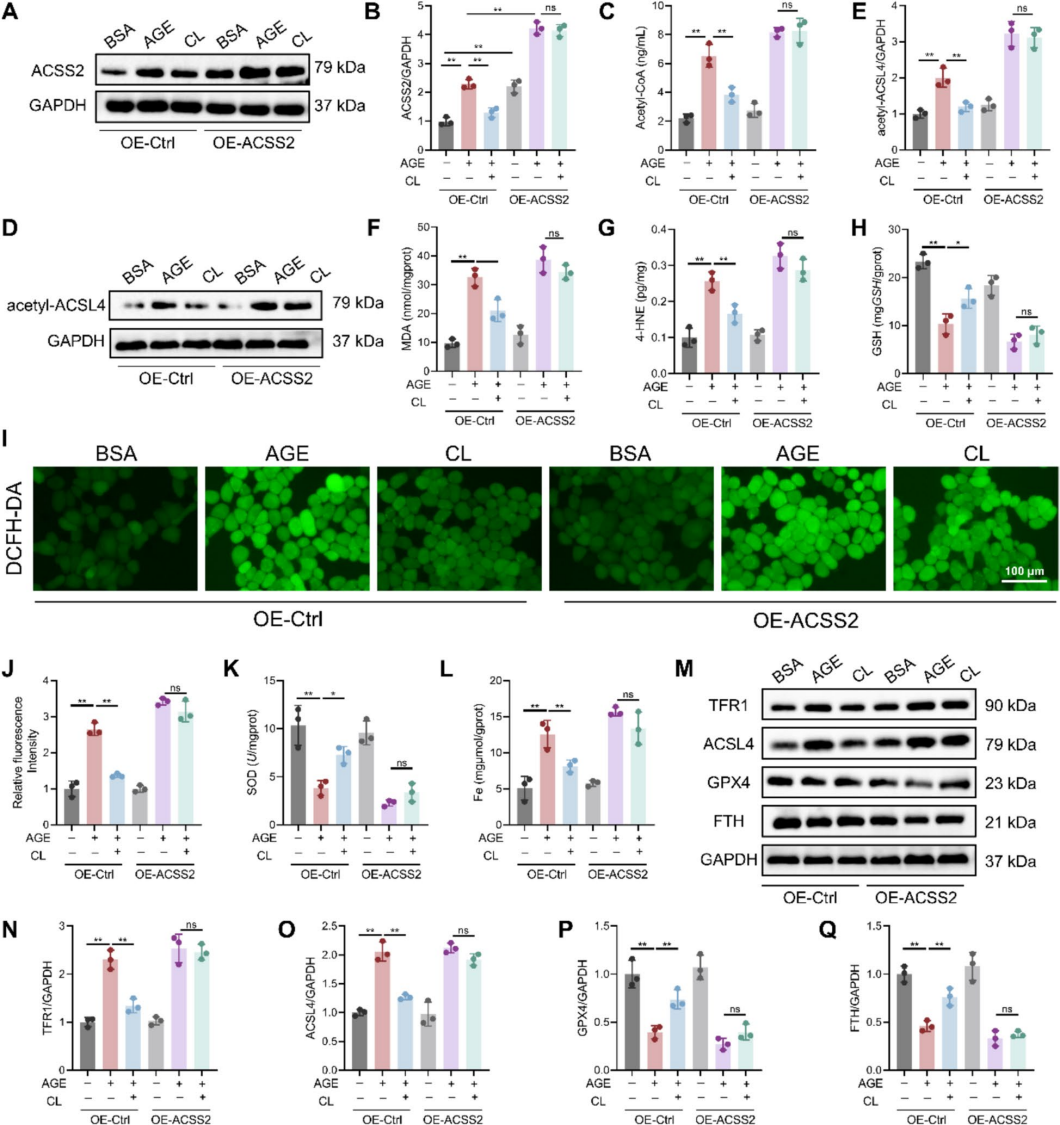
CLY inhibits ACSL4 acetylation through decreasing ACSS2 expression. **A** Representative western blot bands and **B** quantitative analysis for ACSS2 protein in ACSS2-overexpressing HaCaT cells treated with AGEs and CLY, *n* = 3 per group. **C** Intracellular acetyl-CoA content in ACSS2-overexpressing HaCaT cells, *n* = 3 per group. **D** Representative western blot bands and **E** quantitative analysis for acetyl-ACSL4 protein in ACSS2-overexpressing HaCaT cells treated with AGEs and CLY, *n* = 3 per group. **F**–**H** Levels of **F** MDA, **G** 4-HNE, and **H** GSH in HaCaT cells, *n* = 3 per group. **I**, **J**) Representative images of ROS staining using DCFH-DA and corresponding quantitative analysis of fluorescence intensity, *n* = 3 per group. **K** SOD activity and **L** intracellular iron content in HaCaT cells, *n* = 3 per group. **M** Representative western blot bands and quantitative analysis of protein expression levels for **N** TFR1, **O** ACSL4, **P** GPX4, and **Q** FTH in HaCaT cells, *n* = 3 per group. *P < 0.05; **P < 0.01; ns, not significant

Since ACSS2 was the most significantly inhibited by CLY, it was selected for further investigation. As shown in Fig. 8A–C, the protein expression of ACSS2 and the production of acetyl-CoA in HaCaT cells treated with or without CLY were both significantly increased after overexpression of ACSS2. Additionally, the inhibitory effects of acetyl-ACSL4 expression induced by CLY was effectively reversed by ACSS2 overexpression. Similar results were also observed for MDA, 4-HNE, and GSH levels after overexpressing ACSS2, manifested as increased MDA contents, upregulated 4-HNE levels, and reduced GSH levels (Fig. 8D–H). Consistently, elevation of ROS fluorescence intensity and iron levels were observed in CLY treated HaCaT cells following ACSS2 overexpression, whereas exhibited a reduction in SOD activity (Fig. 8I, J). Western blot analysis indicated overexpression of ACSS significantly upregulated the protein expression of TFR and ACSL4, as well as downregulated the GPX4 and FTH levels in AGEs-induced HaCaT cells treated with CLY (Fig. 8M–O). In conclusion, these findings demonstrate that CLY inhibits ACSL4 acetylation through decreasing ACSS2 expression.

### Identification of active ingredients targeting ACSS2 in CLY

**Fig. 9:**
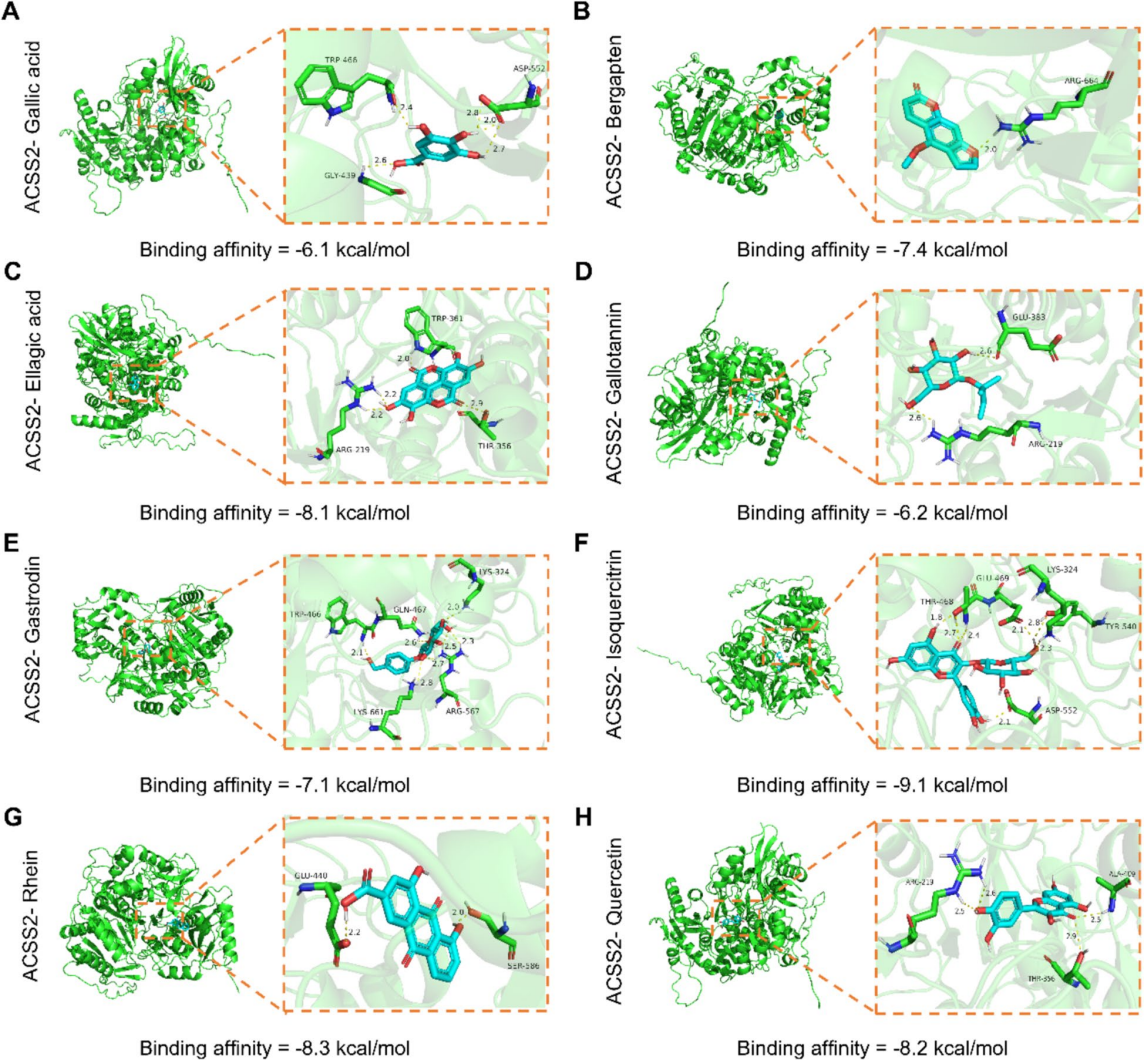
Identification of active ingredients targeting ACSS2 in CLY. **A**–**H** Molecular docking analysis. **A** Gallic acid-ACSS2. **B** Bergapten-ACSS2. **C** Ellagic acid-ACSS2. **D** Gallotannin-ACSS2. **E** Gastrodin-ACSS2. **F** Isoquercitrin-ACSS2. **G** Rhein-ACSS2. **H** Quercetin-ACSS2

To further identify the active compounds in CLY that target ACSS2, molecular docking analysis was performed between the primary chemical constituents and ACSS2. As illustrated in Fig. 9, the binding free energy of Bergapten, Ellagic acid, Gastrodin, Isoquercitrin (IQ), Rhein, and Quercetin with ACSS2 are all below − 7 kcal/mol, suggesting strong binding affinities. Notably, IQ forms hydrogen bonds with ACSS2 residues LYS324, THR468, GLU469, TYR540, and ASP552, indicating a stable and specific interaction. Further in vitro activity evaluation of IQ demonstrated that IQ could inhibited AGE‑induced elevation of acetyl‑CoA levels (Fig. S4A), reduced both ACSL4 protein expression and acetylation (Fig. S4B), and thereby suppressed lipid peroxidation and iron overload in keratinocyte (Fig. S4C and S4D). These findings further validate the molecular docking results and confirm that IQ inhibits ACSL4 acetylation and ameliorates AGE‑induced ferroptosis in keratinocytes.

## Discussion

**Fig. 10:**
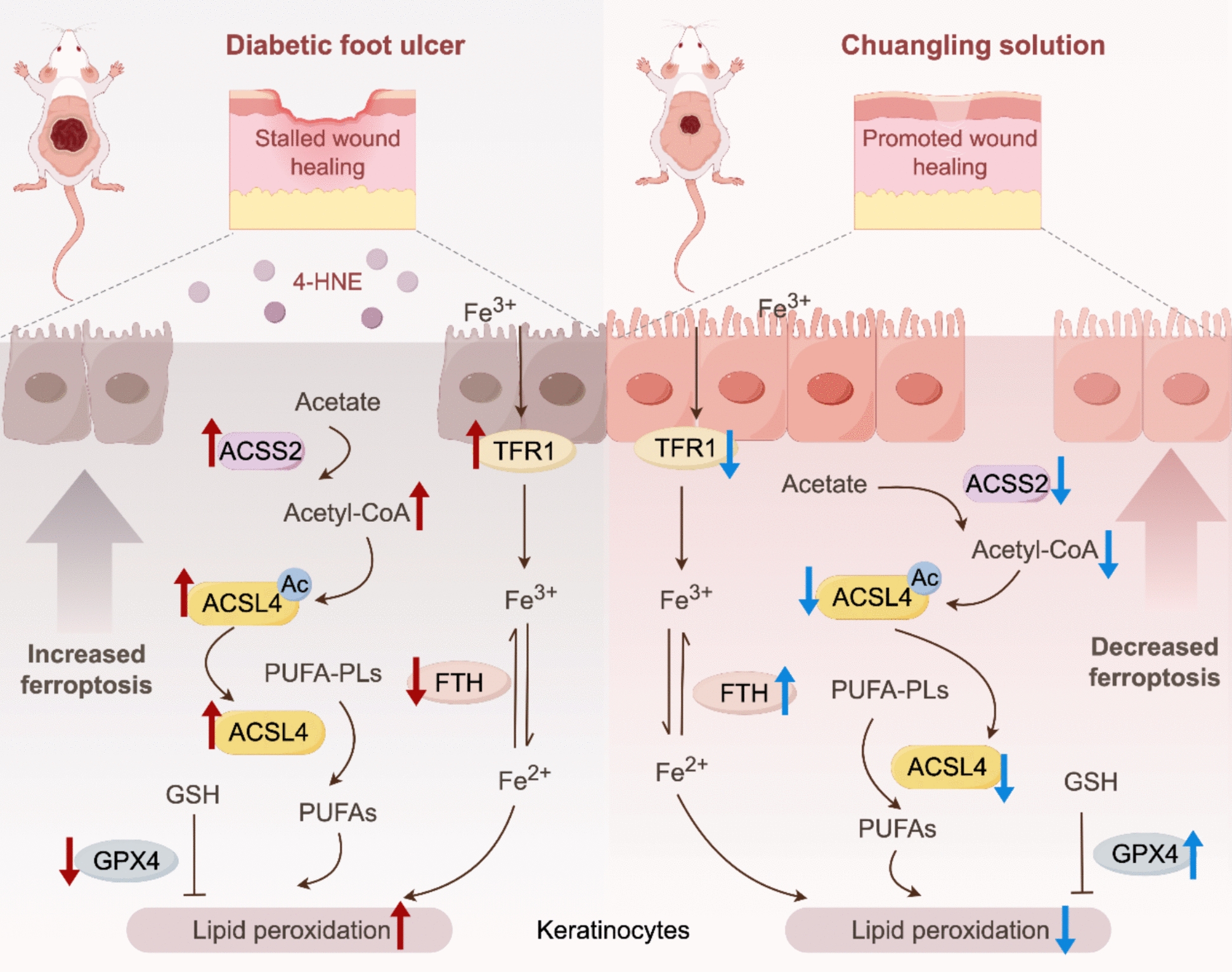
Graphical abstract

DFU is a severe complication of diabetes with high risks of disability and mortality [3]. Due to its complex pathogenesis, the efficacy of clinical interventions remains limited. As a multi-component traditional Chinese medicine formulation, CLY has demonstrated considerable potential in clinical applications for promoting wound healing. This study demonstrated the clinical efficacy of CLY in the treatment of DFU, and revealed that CLY facilitates wound healing under diabetic conditions by inhibiting ACSS2-mediated acetylation of ACSL4, thereby attenuating ferroptosis in keratinocytes (Fig. 10).

Network pharmacology analysis suggests that CLY may exert its therapeutic effects on DFU through multiple potential pathways. Several high-abundance components of CLY identified in this study have also been previously reported to possess bioactivities relevant to diabetes or DFU treatment. Among them, adenosine [15] has been reported to promote angiogenesis and accelerate diabetic wound healing by activating AMPK and upregulating PPARδ. Ellagic acid, a natural polyphenol, can inhibit the formation of AGEs in diabetic animals [32] and enhance the function of dermal fibroblasts and keratinocytes under diabetic conditions via activating EGFR, facilitating collagen synthesis and re-epithelialization [26]. Flavonoids such as quercetin [44], myricetin [39] exhibit broad anti-inflammatory and antioxidant activities. They can ameliorate systemic glucose and lipid metabolic disorders in diabetes, reduce oxidative stress and inflammatory responses, and potentially promote wound healing. Corroborating this literature, the phenotypes of enhanced collagen synthesis and angiogenesis observed in this study support the concept that CLY also mediates pharmacological effects attributable to its active ingredients.

Beyond these multi-component synergistic effects, network pharmacology and transcriptomic analyses specifically highlighted ferroptosis as a critically implicated mechanism in the therapeutic action of CLY. Ferroptosis is closely associated with metabolic processes including iron metabolism, lipid metabolism, and glutathione biosynthesis [43]. In our study, the expression of key proteins involved in these pathways, such as TFR1, FTH, ACSL4, and GPX4, was significantly altered in both in vivo and in vitro models. These changes were markedly reversed following CLY treatment. Previous studies have reported the protective effects of Fer-1, a ferroptosis inhibitor, could attenuate diabetic skin ulcer [17, 18, 38]. Using RSL3 as inducer [4], we found that the beneficial role of CLY in treating skin ulcer in diabetic rats was compromised. These finding suggests that CLY exerts its anti-ferroptosis effect at a more upstream level rather than directly affecting the key molecules involved in the ferroptosis process.

ACSL4, in particular, serves as a critical enzyme linking dysregulated lipid metabolism and ferroptosis. It mediates the transfer of polyunsaturated fatty acids into membrane phospholipids, remodeling the phospholipid composition of cellular membranes and increasing their susceptibility to lipid peroxidation, consequently inducing ferroptosis [5]. This establishes a potential connection between ferroptosis and lipid metabolism, supporting the relevance of the CLY‑regulated lipid metabolism process identified by the GO enrichment analysis. Consistent with our results, previous studies have reported increased ACSL4 expression and lipid peroxide accumulation in the epidermis of diabetic patients and animal models [16, 38]. Notably, knockdown of ACSL4 has been shown to significantly reduce levels of ROS and lipid peroxides, accelerating wound healing in diabetic animals [16]. Furthermore, high glucose conditions can induce upregulation of ACSL4 in keratinocytes, and overexpression of ACSL4 has been found to inhibit keratinocyte viability and migration [38]. These findings highlight the important role of ACSL4-mediated keratinocyte ferroptosis in the pathophysiology of diabetic skin ulcers. In the AGE-induced keratinocyte model utilized in this study, we also observed upregulation of ACSL4 accompanied by typical features of ferroptosis, including iron overload, oxidative stress, and lipid peroxide accumulation. All of these manifestations were attenuated by CLY treatment. These results indicate that the inhibition of ACSL4-mediated ferroptosis in keratinocytes constitutes an important mechanism through which CLY exerts its therapeutic effects on DFU.

In addition to ferroptosis, both the network pharmacology analysis of CLY and the RNA‑seq results of CLY‑treated keratinocytes indicated the potential of CLY to regulate protein phosphorylation biological processes. On the one hand, aberrant protein phosphorylation is involved in the pathogenesis of diabetic skin ulcers. STAT3 phosphorylation is suppressed under high‑glucose conditions, which blocks normal macrophage polarization and delays wound healing [31]. In keratinocytes, high‑glucose stimulation activates ERK1/2, promotes the phosphorylation of c‑Fos and c‑Jun, stabilizes AP‑1, and subsequently upregulates MMP9, leading to collagen degradation [14]. Meanwhile, it has been reported that targeting key regulators or substrates of protein phosphorylation, such as ERK1/2, AKT, and IRS1, can accelerate wound healing under diabetic conditions [10, 35]. On the other hand, the phosphorylation modification of ferroptosis‑related proteins may also participate in the process by which CLY inhibits ferroptosis; for example, the phosphorylation of GPX4 and ACSL4 has been reported to affect ferroptosis in other diseases [36]. Therefore, the significance of CLY‑mediated regulation of protein phosphorylation in its therapeutic effects represents a direction that warrants further mechanistic investigation in future studies.

Impaired wound healing under diabetic conditions results from functional abnormalities in multiple cell types [21], and ferroptosis occurring in various cells also contributes to the pathogenesis of DFU^42^. Fibroblasts is essential for secreting and forming granulation tissue to maintain tissue structure. Previous literature demonstrates downregulation of GPX4 and SLC7A11, both of which are key regulators of glutathione synthesis, along with upregulation of TFR1 under high glucose stimulation [17]. These changes are accompanied by lipid peroxidation and mitochondrial structural damage. Correspondingly, inhibiting ferroptosis in fibroblasts has also been reported to promote wound healing in diabetic animal models [11, 33]. While the present study primarily focused on the significance of CLY in regulating ferroptosis in keratinocytes during DFU treatment, its potential modulatory effects on ferroptosis in other cell types warrant further investigation. For instance, the upregulation of GPX4 and FHT1 in fibroblasts of CLY-treated rat skin suggests that CLY may exert its therapeutic effects by inhibiting ferroptosis in fibroblasts, in addition to its effects on keratinocytes.

We observed that CLY treatment downregulated ACSL4 protein expression without affecting its mRNA level, suggesting that CLY influences the post-translational stability of ACSL4. Transcriptomic analysis of keratinocytes treated with AGEs and CLY revealed that differentially expressed genes were significantly enriched in biological processes related to protein acetylation and acetyl-CoA biosynthesis, revealing an influence on protein function and stability through acetylation modification. Previous studies have demonstrated that acetylated ACSL4 exhibits decreased K48-linked ubiquitination and enhanced protein stability, which in turn aggravates cellular ferroptosis [45]. In AGEs-induced keratinocytes, the acetylation level of ACSL4 was elevated, and proteasome-mediated protein degradation was suppressed, resulting in increased protein half-life. CLY, however, inhibited ACSL4 acetylation and promoted its degradation, thereby reducing ACSL4 protein levels. Transcriptome results indicated that both AGEs and CLY significantly altered the expression of genes involved in acetyl-CoA synthesis, such as *ACSS2* and *ACL solution,* as well as those encoding acetyltransferases and deacetylases including *EP300*, *KAT2B*, and *SIRT1*. These findings suggest that CLY may attenuate ACSL4 acetylation by modulating multiple steps in the protein acetylation pathway. Among these targets, we further investigated the role of ACSS2 in ACSL4 acetylation, as its function in promoting protein acetylation modifications under diabetic complications has been well documented, and its consistent alterations across both animal and cell models provided further rationale for its selection [22, 23]. In this study, we also observed that CLY upregulated SIRT1 expression in keratinocytes. To exclude the possibility that the CLY‑induced reduction in ACSL4 acetylation is mediated by enhanced deacetylase activity of SIRT1, we examined the effect of CLY in SIRT1‑knockdown cells. The results showed that SIRT1 knockdown (Fig. S2A) did not significantly affect the inhibitory effect of CLY on ACSL4 acetylation (Fig. S2B), indicating that the CLY‑mediated suppression of acetylation is independent of SIRT1 upregulation.

In AGE-induced keratinocytes, ACSS2 expression was significantly upregulated, leading to an increase in its catalytic product, acetyl-CoA, which provides the essential substrate for protein acetylation. Meanwhile, previous studies have reported that ACSS2 can inhibit SIRT1 expression and suppress deacetylation activity [22], which suggests a dual role of ACSS2 in promoting protein acetylation. Notably, our study also shows that CLY inhibits acetylation of ACSL4 in a SIRT1-indepenent way. Numerous studies have reported that several natural SIRT1 activators serve as candidate therapeutic molecules for promoting diabetic wound healing [25], while the focus on ACSS2 remains relatively limited. Artificial overexpression of ACSS2 in cells would theoretically attenuate or even abolish the inhibitory effect of CLY on ACSS2 expression, allowing us to examine whether the effects of CLY depend on downregulating ACSS2 expression. In this study, overexpression of ACSS2 in keratinocytes attenuated the beneficial effects of CLY on inhibiting of ACSL4 acetylation, oxidative stress and lipid peroxidation. These findings demonstrate that ACSS2 is a critical target through which CLY ameliorates ferroptosis in keratinocytes under diabetic conditions and facilitates wound healing. Based on this, IQ was identified from CLY as a potential ACSS2 inhibitor with validated effects in inhibiting ACSL4 acetylation and thereby suppressing ferroptosis in vitro. While its in vivo activity and precise molecular mechanism interplaying with ACSS2 needs further investigation.

The significant role of ACSS2 upregulation in renal tubular epithelial cells in promoting glucolipid metabolic disorders and aberrant protein acetylation has been established in the pathogenesis of diabetic nephropathy [22, 23], and the protective effect of ACSS2 knockout on renal function has been experimentally confirmed [23]. Our study extends the relevance of this metabolism-associated target, ACSS2, to DFU, and underscores its potential as a therapeutic target shared across multiple diabetic complications. Additionally, the other active components in CLY responsible for inhibiting ACSS2 expression require further elucidation.

## Conclusion

In conclusion, this study confirms the clinical efficacy of CLY in the treatment of DFU. Through both in vivo and in vitro experiments, we demonstrate that CLY inhibits ferroptosis in keratinocytes by modulating the ACSS2 mediated acetylation of ACSL4. These findings elucidate the mechanism by which the traditional Chinese medicine formulation CLY promotes diabetic wound healing and highlight the significance of its key target, ACSS2, as a potential therapeutic target for this condition.

## Supplementary Information

Additional file1 (DOCX 820 KB)

## Data Availability

No datasets were generated or analysed during the current study.

## Abbreviations

ACSL4: Acyl-CoA synthetase long chain family member 4
ACSS2: Acyl-CoA synthetases short chain family member 2
AGEs: Advanced glycation end products
CHX: Cycloheximide
CLY: Chuangling Ye
DEGs: Differentially expressed genes
DFU: Diabetic foot ulcers
DL: Drug-likeness
Fer-1: Ferrostatin-1
GO: Gene ontology
GSH: Glutathione
KEGG: Kyoto Encyclopedia of Genes and Genomes
KCs: Keratinocytes
MDA: Malondialdehyde
OB: Bioavailability
PFA: Paraformaldehyde
rb-bFGF: Recombinant bovine basic fibroblast growth factor
SOD: Superoxide dismutase
STZ: Streptozotocin
TEM: Transmission electron microscopy
VEGF: Vascular endothelial growth factor
4-HNE: 4-Hydroxynonenal
