# Supplementary material for: Chuangling Ye mitigates diabetic foot ulcer through suppressing keratinocyte ferroptosis via inhibiting ACSS2/ACSL4 axis
Source: Chin Med. 2026 Jun 8;21:162. doi: 10.1186/s13020-026-01435-8 (PMC13244978; doi:10.1186/s13020-026-01435-8)
Supplement: Supplementary file 1 — Additional file1 (DOCX 820 KB) [file 13020_2026_1435_MOESM1_ESM.docx]

Dear editors of ***Chinese medicine***

I am writing to respectfully request a correction regarding the article type of our manuscript, “*Chuangling Ye mitigates diabetic foot ulcer through suppressing keratinocyte ferroptosis via inhibiting ACSS2/ACSL4 axis*” (DOI: 10.1186/s13020-026-01435-8), currently at the proof stage.

Upon careful review of the proof, it has come to our attention that the article type was incorrectly designated as "Brief Report" at the time of submission. The manuscript was conceived, structured, and written in full accordance with the format and scholarly scope of a standard Research Article. This misclassification was an inadvertent oversight on our part, and we sincerely regret any confusion or inconvenience it may have occasioned.

We would be most grateful if the editorial office could kindly facilitate the change of the article type from "Brief Report" to "Research Article." Please do not hesitate to let us know should any further information or procedural steps be required.

Thank you for your kind consideration and assistance.

Yours sincerely,

Chaoqun Ma, Affiliated Hospital of Nanjing University of Chinese Medicine,

Nanjing, 210029, China

E-mail: [szpumcq@sina.com](mailto:szpumcq@sina.com)
